# Passing Topics and Movements

**Published:** 1918-06-29

**Authors:** 


### THE WOMAN WORKER'S WORLD.

### Passing Topics and Movements.

A RECENT correspondence in the *Yorkshire Post* shows the fatal ease with which misunderstandings can grow and be disseminated. The excellent scheme of Judge Neil, which he began in Chicago, and which the American States are one after another taking up, is not one for the general endowment of motherhood. That is quite another question; it is the financing of widows and deserted wives, whose children must either suffer want or come upon the rates, which is arranged. The breaking up of families by the planting out in different institutions of the various children was easily shown, once public attention was turned to it, to be an economic as well as a social crime and folly. A very important feature of the scheme is that the mother is regularly visited, and the reports show that in the vast majority of cases the public money given till the children are able to earn is conscientiously managed. Infant welfare work in Germany is on wrong lines because the child is regarded almost exclusively as the citizen, and not as the individual and member of the family. Judge Neil and those who think with him believe that to increase the number of happy homes is worth every effort, and that no woman who should be home-maker can successfully attempt to be simultaneously bread-winner.

BUT as things are, the task of some women as home-makers needs to be made possible. Recently on a hot afternoon the present writer sat with a woman in one of the two rooms in which her family of seven, or eight when the husband returns, have their being. There was a fire in the pitiful "bedroom" grate, where she must cook, the company will not put in a gas-stove till after the war. Better rooms are hard to obtain. People do not want large families as tenants. The walls are never free from vermin, despite the poor woman's efforts to be clean. And vermin mean troubled sleep for children already suffering from too close quarters.

THE relation of this housing question to tuberculosis was raised again at a discussion at the Women's Institute on June 11, when Mr. Brown, chairman of the Workmen's Housing Association, found serious fault with the cottage designs of members of the Institute of British Architects. The living-room in these plans contained too few cubic feet of air for the health of a family. The domestic architect certainly needs to give more consideration to the question of the family wash. In Scotland a washhouse is far more commonly considered a necessity of life than it is in this country; in the case of tenement dwellings three or four families would share one; in the country a shed in the garden is desirable. How many babies, we may ask, in the cities die yearly from bronchitis as the result of steam from wash-tubs and drying clothes? Where public baths offer laundry convenience these are eagerly competed for; mothers are well aware of the dangers involved.

**The Woman Worker's World**—(*continued*).

LORD BURNHAM and Dr. Truby King gave addresses recently to a conference of social workers interested in emigration for women, both emphasising the fact that the Dominions greatly resent any influx of unskilled industrial workers, but that for the truly competent home-maker there is any amount of demand. Farmers in New Zealand, Dr. Truby King said, are surprised that more girls are not willing to come out, become one of the family, and marry amongst their new friends. But of fifty emigrants who crossed with him nine were teachers going to posts; of the remainder not one was fit to undertake household work. Lady Knox spoke of openings for trained nurses in Rhodesia, living in hostels and going out to cases, beginning at £72 salary, and passage paid both ways for two years' efficient work.

THERE is always consolation to human nature in being told that others have done worse in a matter in which we deserve blame. Our infant mortality rate has been shown to be a disgrace by the rapid drop which follows remedial measures in the ratio of their vigour. That of Germany was nearly 50 per cent. higher at the close of the last century. In 1901 it was 151 per thousand, in 1913 it was 108, and the reduction, as we know, continues. In Germany the rate of 207 in 1901 had only been reduced to 151 by 1913. We need not think of resting on these laurels; the ideal still looms afar of a time when no mother's travail shall needlessly be wasted, and no little citizen be handicapped for life by early mismanagement. It is interesting to note that in Dantzig the giving of the Imperial grant in 1915 to soldiers' wives brought no improvement. But when in January 1916 a Welfare Centre was started, fifteen months' work raised the number of breast-fed infants from 40 to 60 per cent., and lowered the mortality of legitimate children from 177 to 109.

THE Intelligence Department of our own Local Government Board has published a digest of a number of German reports at 6d. This is full of interesting facts, and may be had through any bookseller. Bavaria has a great pioneer in infant welfare in Dr. Eidam, medical officer of Gunzenhausen, who perceived that the country doctor's best chance of influence with a mother was the visit for vaccination. He arranged that each doctor of his district should on this occasion fill up a form showing how each child was fed, and forward it to him, and he persuaded the district authorities to pay a small fee for visits by midwives till each child was nine months old. Seven years of this work have lowered the local death-rate and raised the number of breast-fed babies from 60 to 80 per cent. Throughout the Empire efforts are being made to mitigate the sufferings of mothers and infants through shortage of food, especially of milk and fats.

WE might profit by the idea started at Berlin in 1914 by a Red Cross Committee, that of "War Sponsors," who would take individual care of needy mothers and babes. At Dresden 300 people at once responded to the appeal, and a report from Mannheim relates that sixty persons had cared for 119 families, and that beneficial results appeared. In view of the fact that the opinion in Prussia is that peasant women would receive with mistrust advice from women of the property-owing classes, and that in Bavaria, which has a higher mortality rate than Prussia, it is the popular belief that many infants must die early, education is plainly the great need. In this country we are divided between the opinion that mothers above all need knowledge, and the view that only stress of circumstances keeps them from practising what they well understand.

SIR WILLIAM TRELOAR has taken a valuable step forward in the care of crippled children and their education as wage-earners by instituting special post-graduate courses for doctors at Alton. Nearly five times as many as could be accommodated applied for the first course in April under Dr. Gauvain, a truly encouraging response. This hospital has now eight certificated women teachers under the Board of Education, turning the atmosphere of "listless indifference" into one of "joy, cheerfulness, and laughter." Londoners who would like to see the effect of "lessons" (we have to use the word) on sick—sometimes very sick—children, could not do better than ask permission to visit the L.C.C. Special School, Cromwell House, Highgate Hill, N. One of the reconstructive works for which money will be needed after the war will be the founding of more residential schools for the crippled child.

